# Comment on “Effect of maternal cortisol levels on fetal heart rate patterns in primiparous pregnant women in the third trimester”

**DOI:** 10.1590/1806-9282.20231300

**Published:** 2024-02-26

**Authors:** Mingyun Cheng, Yuping Duan

**Affiliations:** 1 Shiyan Hospital of Traditional Chinese Medicine, Maternity Department - Shiyan, China.; 2 Shiyan Maternal and Child Health Hospital, Maternity Department - Shiyan, China.

Dear Editor,

Turan and Kaya’s^1^ recent study explores a critical aspect of pregnancy, focusing on the impact of maternal cortisol levels on fetal heart rate patterns among primiparous pregnant women in the third trimester. Their research provides valuable insights into the complex relationship between maternal stress and its potential consequences for fetal well-being. In this study, Turan and Kaya^1^ investigated the correlation between maternal cortisol levels, a hormone linked to stress, and fetal heart rate patterns. Their findings highlight the significance of monitoring and managing maternal stress during pregnancy, as elevated cortisol levels are associated with alterations in fetal heart rate patterns. These results prompt important questions regarding the potential long-term effects of stress on fetal development. Furthermore, Turan and Kaya meticulously analyzed data from a cohort of primiparous pregnant women, offering a comprehensive understanding of a demography often underrepresented in research. This inclusivity enhances the applicability of their findings and underscores the necessity for a broader perspective on the intricate factors influencing fetal health. However, some specific concerns require further clarification.

First, it is important to note that serum cortisol levels may provide a more accurate reflection of maternal cortisol levels compared to cortisol levels in saliva. As demonstrated in this study^1^, a moderately positive relationship between fetal heart rate and maternal salivary cortisol levels was observed (r=0.448, p=0.000). However, the correlation coefficient of 0.448 between fetal heart rate and maternal salivary cortisol levels suggests a relatively weak association. This could potentially be attributed to the fact that cortisol levels in saliva may not authentically represent maternal cortisol levels. It is plausible that replacing cortisol levels in saliva with serum cortisol levels could reveal a stronger correlation between cortisol levels and fetal heart rate. Consequently, using salivary cortisol levels to represent maternal cortisol levels in this study may have underestimated the relationship between cortisol levels and fetal heart rate. Moreover, considering that obtaining blood samples from pregnant participants in this study is not inherently challenging, it would be worthwhile to further investigate the relationship between serum cortisol levels and fetal heart rate. This could potentially yield more precise evidence, enhancing our understanding of this association.

Second, as the primary objective of this study^1^ is to explore the impact of maternal cortisol levels on fetal heart rate patterns, it is essential to emphasize that numerous factors are associated with fetal heart rate patterns, not solely maternal cortisol levels. Research by Monk et al.^2^ has indicated that pregnant women with comorbid depression and anxiety exhibit a greater increase in heart rate compared to controls. In addition, Monk et al.^2^ also found a significant correlation between paced breathing and fetal heart rate. The evidence suggests that salivary cortisol levels are not the sole influencing factor, and maternal breathing rate and psychiatric status have also been demonstrated to have a significant relationship with fetal heart rate. However, this study^1^ lacks detailed information regarding maternal breathing rate and psychiatric status. One plausible hypothesis is that fetal heart rate is an outcome influenced by the combined effects of these three factors, rather than being determined by maternal cortisol levels alone. In light of these considerations, it is important to recognize that a more comprehensive approach may be needed to fully elucidate the complex interplay of factors contributing to fetal heart rate patterns in primiparous pregnant women in the third trimester. Further investigations into these additional variables could provide a more nuanced understanding of the observed correlations.
